# Porous Ultra-Thin Films from Photocleavable Block Copolymers: In-Situ Degradation Kinetics Study of Pore Material

**DOI:** 10.3390/polym12040781

**Published:** 2020-04-02

**Authors:** Sedakat Altinpinar, Wael Ali, Patrick Schuchardt, Pinar Yildiz, Hui Zhao, Patrick Theato, Jochen S. Gutmann

**Affiliations:** 1 Institute for Physical Chemistry, University of Duisburg-Essen, 45141 Essen, Germany; ali@dtnw.de (W.A.); patrick.schuchardt@uni-due.de (P.S.); 2 Deutsches Textilforschungszentrum Nord-West gGmbH, 47798 Krefeld, Germany; 3 Institut für Energie- und Umweltverfahrenstechnik, University of Duisburg-Essen, 45141 Essen, Germany; pinar.yildiz@uni-due.de; 4 Institute of Fundamental and Frontier Sciences, University of Electronic Science and Technology of China, Chengdu 610000, China; huizhao.com@gmail.com; 5 Institute for Chemical Technology and Polymer Chemistry, Karlsruhe Institute of Technology (KIT), 76131 Karlsruhe, Germany; patrick.theato@kit.edu; 6 Institute for Biological Interfaces III, Karlsruhe Institute of Technology (KIT), 76344 Eggenstein-Leopoldshafen, Germany; 7 CENIDE, University of Duisburg-Essen, 47057 Duisburg, Germany

**Keywords:** block copolymers, photocleavage, in-situ SFM, in-situ X-ray reflectivity, GISAXS

## Abstract

On the basis of the major application for block copolymers to use them as separation membranes, lithographic mask, and as templates, the preparation of highly oriented nanoporous thin films requires the selective removal of the minor phase from the pores. In the scope of this study, thin film of polystyrene-*block*-poly(ethylene oxide) block copolymer with a photocleavable junction groups based on ortho-nitrobenzylester (ONB) (PS-*hν*-PEO) was papered via the spin coating technique followed by solvent annealing to obtain highly-ordered cylindrical domains. The polymer blocks are cleaved by means of a mild UV exposure and then the pore material is washed out of the polymer film by ultra-pure water resulting in arrays of nanoporous thin films to remove one block. The removal of the PEO materials from the pores was proven using the grazing-incidence small-angle X-ray scattering (GISAXS) technique. The treatment of the polymer film during the washing process was observed in real time after two different UV exposure time (1 and 4 h) in order to draw conclusions regarding the dynamics of the removal process. In-situ X-ray reflectivity measurements provide statistically significant information about the change in the layer thickness as well as the roughness and electron density of the polymer film during pore formation. 4 H UV exposure was found to be more efficient for PEO cleavage. By in-situ SFM measurements, the structure of the ultra-thin block copolymer films was also analysed and, thus, the kinetics of the washing process was elaborated. The results from both measurements confirmed that the washing procedure induces irreversible change in morphology to the surface of the thin film.

## 1. Introduction

**Scheme 1:**
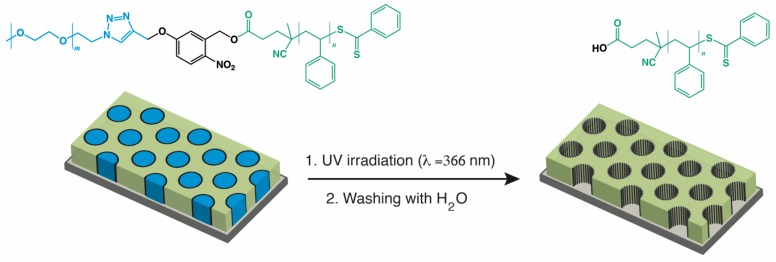
Schematic representation of photocleavable block copolymer thin film and the subsequent removal of the minor block upon photocleavage and washing.

The production of nanoporous materials have attracted tremendous interest due to their immense application in variety of fields, including separation, catalysts, biomaterials engineering, and electronics [1,2,3]. Many methods have been used for producing such nanoporous with controlling the pore structures. For instance, CO_2_ foaming agent has been used to prepare nanoporous polymer materials, including nanocellular and thin polymer films [4,5,6]. Another approach for fabricating nanoporous materilas is templating techniques [7]. Highly-ordered thin films is one of the outstanding fields of research in the context of block copolymers since they enable a versatile self-assembled morphology in the range of 5–50 nm by means of a “bottom-up” approach [8,9,10]. They promise applications, such as polymer membranes or templates for nanostructured materials [11,12,13,14,15,16]. Among the block copolymer structures, vertical cylinders have received great attention because of their ability to produce highly ordered nanopores [8,17]. Nanopores can be formed from a thin block copolymer film by removing one block by selective etching or by dissolving one polymer block [18,19,20]. To date, several methods for the removal of one block out of a thin film have been reported, such as chemical etching, ozonolysis, hydrolysis, and UV degradation. Overall, they can only be realized under harsh treatment conditions [21,22,23]. When compared to these methods, photolabile junction groups that are based on ortho-nitrobenzylester (ONB) were introduced to cleave the two blocks under mild conditions [24,25,26]. Light provides a particularly powerful and multilateral stimulation because it can be localized both spatially and temporally, and additional reagents are not needed. The properties of ordered polymers in thin films significantly differs from the properties of the bulk system, as enthalpic and entropic effects influence the behavior of the polymer. When the bulk polymer is exposed to a good solvent for the corresponding polymer, the solvent molecules will enter the free volume of the polymer, resulting in the swelling of the polymer coils. However, in thin polymer films, the swelling dynamics are very sensitive to the changes in interaction within the system. This plays an important role in the washing out of the minor component for the production of porous thin films. In this work, polystyrene-*block*-poly(ethylene oxide) diblock copolymer is used with ortho-nitrobenzyl ester (ONB) as a photocleavable junction linking the two blocks (PS-*hν*-PEO). A suitable solvent washes out the pore forming material PEO after the two blocks are photocleaved by applying mild UV radiation (see Scheme 1).

The stability of ultra-thin block copolymer films and their morphology was investigated. For this purpose, in-situ X-ray reflectivity (XRR) and in-situ scanning force microscopy (SFM) measurements were carried out to observe the film behavior in real-time. In addition, grazing incidence small-angle X-ray scattering (GISAXS) was also performed to obtain information regarding the lateral structure that is perpendicular to the film surface. In a GISAXS scattering geometry, the X-ray beam is directed onto the sample at a small angle of incidence that is close to the critical angle of total reflection of the used material resulting in a two-dimensional (2D) GISAXS scattering image is recorded with a 2D detector [27]. 

XRR is a very informative non-destructive method for measuring film thickness and electron density perpendicular to the sample surface [28]. In addition, it provides precise information regarding the film roughness, which can be compared with the results from SFM measurements [29,30]. SFM measurements studied the influence of the kinetics on the surface structure during the washing process. The understanding of the mobility of the polymer chains in ordered geometry and their equilibrium in the presence of a solvent is of technological importance. Moreover, the swelling dynamics and swelling kinetics are of great importance from a scientific point of view for the understanding of such systems.

## 2. Materials and Methods 

### 2.1. Thin Film Preparation

A polystyrene-*block*-poly(ethylene oxide) block copolymer with a photocleavable ortho- nitrobenzyl ester junction (PS-*hν*-PEO) was synthesized while using a combined reversible addition-fragmentation chain-transfer (RAFT) polymerization and “click chemistry” approach, yielding a molecular weight of M_W_ = 27000 g/mol for PS and M_W_ = 5000 g/mol for PEO (D = 1.22 for the total block copolymer) [24]. Silicon wafers were used as substrates. The wafers were cut in 2 cm × 2 cm pieces and then cleaned in O_2_-plasma for 15 min. at a pressure of 0.6 mbar and a plasma power of 100 W. A 0.3 nmol/L stock solution of polymer in toluene was stirred overnight and filtered before the spin coating of the thin films. For each sample, 100 μL of the polymer solution was spin-coated onto a cleaned silicon wafer while using 3000 rpm for 60 s. Well-ordered polymer thin films were prepared after solvent annealing with H_2_O/THF for 2.5 h. Photocleavage of the polymer films using UV irradiation was conducted at a wavelength of 366 nm for 1 h and 4 h with a UV Hand Lamp (Herolab GmbH, Wiesloch, Germany) of 6 W power. All of the solvents were of analysis grade, unless otherwise noted. 

### 2.2. In-Situ X-ray Reflectivity (XRR) Experiments

The X-ray reflectivity measurements were performed at the Bl9 beamline at the synchrotron light source DELTA in Dortmund, Germany [31,32,33,34]. We used hard X-rays with a photon energy of 27 keV (0.459 Å) to penetrate the liquid area without loss. The beam size was 0.2 mm × 1 mm (vertical × horizontal). The reflected intensity was measured by the NaI point detector in a distance of 1 m from the sample. A sample cell is used for the investigation of the solid-liquid interface, which had two Kapton windows for beam entrance and exit. The incident X-rays pass the Kapton windows close to 90°. The sample was placed in a sample carrier to permit a stable position of the wafer during the measurement. 

The sample was first aligned in *n*-heptane and then the first scan in $q_{z}$ range of 0.01–0.42 Å^−1^ was recorded Å^−1^ in order to record the full kinetics. Afterwards, ultra-pure water was added into the sample cell and a scan in smaller $q_{z}$ range of 0.01–0.167 Å^−1^ was repeated until reaching the time scale, where we expected the process to be completed. Thereafter, a full scan was again performed in the range of 0.01–0.42 Å^−1^. X-ray reflectivity studies on air-solid interfaces were performed prior to in-situ measurements as well as after it.

For the analysis, the scans in air before and after washing were fitted while using the Parratt algorithm. The reflectivities were multiplied by $q_{z}^{4}$, which compensates the strong decrease of the reflected intensity, to distinguish and compare the oscillation periods.

### 2.3. In-Situ Scanning Force Microscopy Measurements

The SFM measurements were performed with the Agilent Technologies SFM 5500 system that was operated in tapping mode. Ex-situ measurements were carried out in air using cantilevers from Nanosensors with silicon tips with a force constant 21–98 N/m and a resonance frequency of 146–236 kHz. The resolution of the scans was set at 1024 data points per 1 μm and the scan speed was 0.5 lines per second.

The in-situ SFM imaging was performed in the liquid cell. A NanoWorld triangular shaped silicon nitride cantilever with a force constant of 0.32 N/m was used. Before the real measurement, a comparable sample was measured in ultra-pure water to align the cantilever to the actual force constant (99.61 kHz) in liquid and, thus, to save time in the real time imaging. After the alignment, the real sample was insert into the liquid cell and then immersed with ultra-pure water. An area of 1 × 1 μm^2^ was scanned with a resolution of 1024 data points and a scan speed of 0.8 lines per second for the trace scan and 0.5 lines per second for the retrace scan in y-direction.

The pico image software was used for analysis in terms of height distribution and bearing ratio. The roughness evaluation for the different selected areas on the images in liquid and the fast Fourier transform (FFT) calculations were undertaken with the software Gwyddion.

### 2.4. Grazing Incidence Small-Angle X-ray Scattering Measurements

The GISAXS measurements were conducted at the at the SAXS/WAXS/GISAXS beamline 7.3.3 of Advanced Light Source (ALS) synchrotron at Lawrence Berkeley National Laboratory in Berkeley, CA, USA [35]. The beam size was 30 μm (V) × 50 μm (H), flux = 10 E12, Monochromator dE/E = 0.01. The sample-to-detector distance was 3780 mm calibrated while using silver behenate. An angle of incidence of α_i_ = 0.4° was utilized for the polymer thin films, which is above the critical angle of total reflection of PS and silicon. The synchrotron energy was 10 keV (a wavelength of λ = 0.124 nm). For detecting the scattered x-ray signal, the Pilatus 300KW detector was used with 1475 × 1679 pix^2^ and a pixel size of 172 × 172 μm^2^. The out-of-plane scan cuts were performed along the wavevector $q_{y}$ direction on the 2D GISAXS patterns to extract one-dimensional (1D) scattering profiles in order to detect the formation of nanodomains of the photocleaved polymer thin films and the distance between the cylindrical PS domains. The data have been analysed using the programs FitGISAXS integrated in IgorPro [36].

## 3. Results and Discussion

### 3.1. In-Situ X-ray Reflectivity

The film thickness and roughness were investigated using in-situ XRR measurements to pursue the question if the thickness of the film changes during the washing process and whether this has an effect on the film thickness after washing in dry state. With regard to roughness, a change after washing is to be expected, if PEO could be washed out of the pores. In this case, the reflectivity curves were fitted using the Parratt algorithm, whereby we do not focus on the quantitative, but on the qualitative result. This allows for the comparison of the results with those of the SFM measurements. In addition, the fits give values for the electron density of the material. Neutron scattering measurements are required in addition in order to be able to draw a reliable conclusion for the composition of the pores. These, in turn, do not allow for measurements in real time. The temporal resolution of the measurements is important since the investigation of the system focuses on the kinetics of the swelling process. X-ray reflectivity measurements give very accurate values over the layer thickness in the minute range, which can be determined without a fit and it can be calculated over the oscillation minima or maxima. 

**Figure 1:**
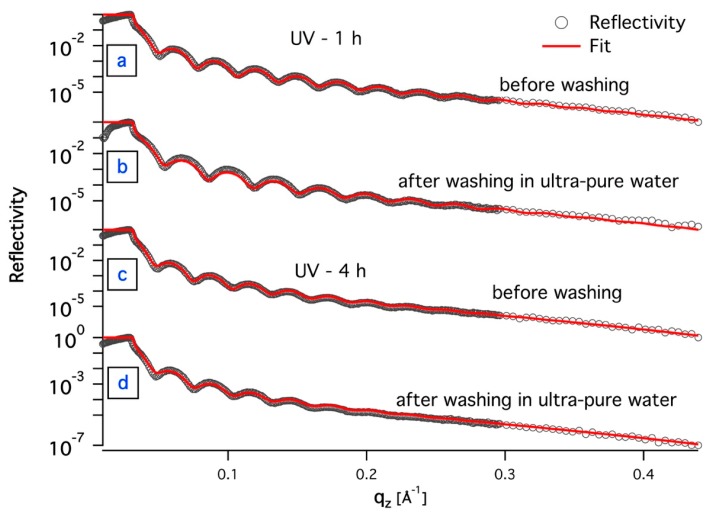
X-ray reflectivity curves of photocleavable junction linking the two blocks (PS-*hν*-PEO) ultra-thin film measured in dry state. The reflectivities for the sample cleaved with UV for 1 h are represented in (**a**) before and (**b**) after washing out the pore material with ultra-pure water. The reflectivities for the sample cleaved with UV for 4 h are represented in (**c**) before and (**d**) after washing out the pore material with ultra-pure water. The black line represents the measured curve and the red line is the fit simulated using the Parratt algorithm.

Two different samples were measured following the same procedure, namely a sample that was irradiated with UV for 1 h and the another was irradiated for 4 h. For both samples, a reflectivity curve was recorded in dry state before and after the in-situ measurement, as shown in Figure 1 with the corresponding fits. The reflectivity curves were normalized to the highest intensity.

As a first step of the in-situ washing measurements, the samples were aligned in *n*-heptane. PS and PEO both have a repulsive interaction with *n*-heptane, since the Flory-Huggins interaction parameter *χ* takes the positive value of 1.13 for PS and 7.21 for PEO [37]. Hence, no interaction between the film and *n*-heptane occurs, enabling a sample alignment without PEO removal. *n*-heptane has a lower density than water and, hence, is forced upwards away from the sample after the addition of water into the liquid cell. A full X-ray reflectivity curve was recorded in *n*-heptane before the start of the swelling.

**Figure 2:**
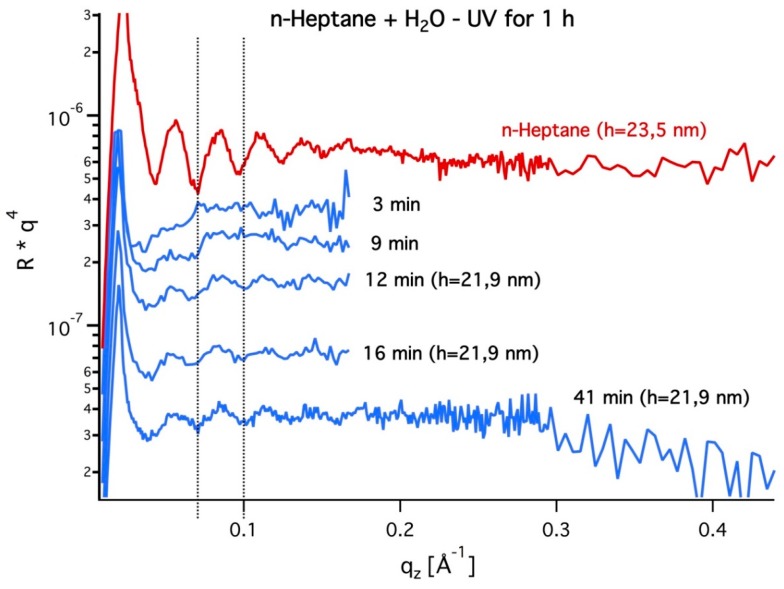
In-situ X-ray reflectivity curves of PS-*hν*-PEO ultra-thin film UV-cleaved for 1 h during washing out the pore material. The red curve represents the sample in n-heptane for sample alignment. The blue reflectivity curves correspond to the measurements in ultra-pure water with the according washing time and film thickness h. The curves are plotted as $R*q^{4}$ versus $q_{z}$ to eliminate the strong drop of the reflectivity and to enhance the visibility of the Kiessig fringes.

It is noticeable that the curves depicted in Figure 1 have discrepancies with the fit in the region below the critical angle. This is due to the fact that at small angles of incidence the footprint of the beam over illuminates the sample. With an increasing angle of incidence, the Kiessig fringes form, from which the information normal to the sample surface can be obtained. The reflectivity curve for the sample cleaved for 1 h with UV before washing (Figure 1a) yields a layer thickness of 21.9 nm and roughness of 6.7 Å. After adding *n*-heptane into the sample cell the film thickness increases to h = 23.5 nm, so the film swells by about 10%, as shown in Figure 2 (red curve). As *n*-heptane is a non-polar solvent and does not have the ability to form hydrogen bonds, this behavior can be attributed to the repulsive forces between the film and the solvent. The thin film is forced to contract in the vertical direction, since the space is restricted in the lateral and perpendicular directions.

This can be derived from the fact that the process is reversible. After the addition of water, the polymer film relaxes, and the thickness of the polymer film decreases again. The blue curves in Figure 2 show the measured curves after the addition of water into the liquid cell. The oscillations are again visible after *n*-heptane is forced out of the sample surface by water, and a film thickness of 21.9 nm can be calculated. In the further progress of the measurement in water, the film thickness does not change any longer. Regarding the change in the roughness and the scattering density, one can be geared to the oscillations, whereby the values cannot be determined in the plot. Nevertheless, in the present measurement the Kiessig fringes give indication that a dissolution process can be observed around 12 min. in water. Washing after 16 min. shows a weakening of the last oscillations in the curve when compared to the measurement before. From this, it could be inferred that after 12 min. immersing the film in water, the pore material PEO diffuses onto the sample surface, thereby altering the roughness at the polymer film-water interface and the electron density within the film. In the measurement after 41 min., where the scan was taken over a larger angular range, it can be seen that the curve has comparable oscillations at higher $q_{z}$ values as in the measurement after 12 min. in water. From this result, a substantial change in the roughness would not be expected for the sample. This indicates that the pore material could not be washed out. In order to make a clear statement, whether PEO is still present in the sample, the sample was dried in air for 2 h and then scanned again in dry state.

Figure 1b shows the reflectivity curve of the sample after washing and drying with the corresponding fit. The evaluation of the reflectivity yields a value of 19 nm for the layer thickness and a roughness of 6.8 Å. Table 1 summarizes the data that were obtained from the XRR measurements before and after the washing of the sample. It becomes clear that the film thickness after washing is reduced by 2.9 nm, but the roughness has not clearly changed.

**Figure 3:**
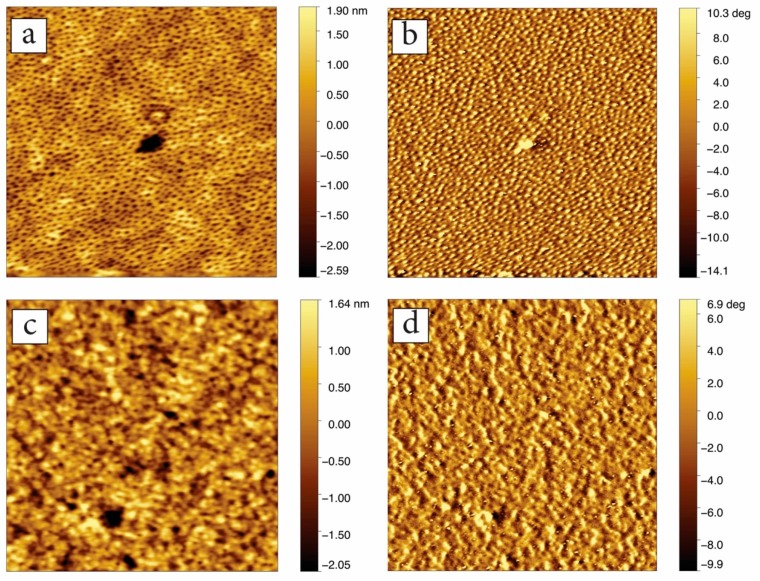
Scanning force microscopy (SFM) topography (**a**,**c**) and phase (**b**,**d**) images (1μm × 1μm) before (**a**,**b**) and after (**c**,**d**) in-situ measurements with ultra-pure water upon UV cleavage for 1 h.

The change in film thickness can be attributed to the interaction of PEO with the substrate while assuming that the block copolymer has not been cleaved under UV radiation for 1 h. The hydrophilic pore material still bounded covalently to PS is partially moved onto the film surface after immersing in the solvent. The whole film is contracted due to the attractive force between the PEO and the likewise hydrophilic substrate, resulting in the decrease in the layer thickness. The sample was proved for the surface structure with SFM before and after the XRR measurement to verify this process. Figure 3 shows the topography and phase images of the sample.

**Figure 4:**
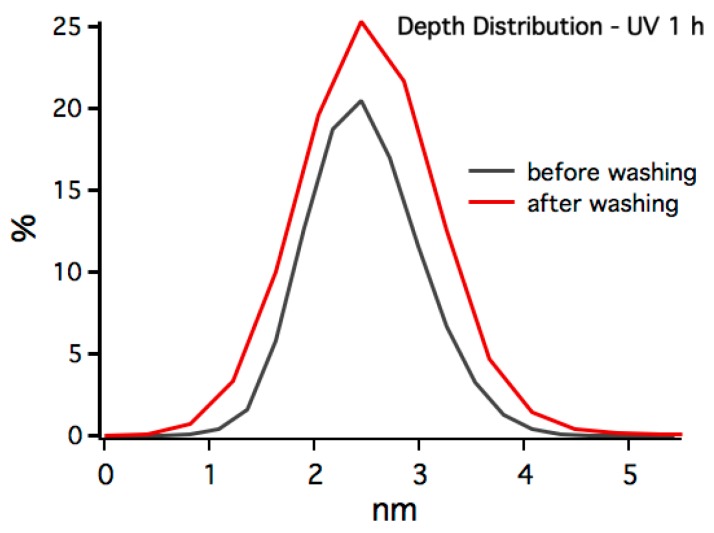
Depth distribution evaluated from the SFM images (Figure 3) before (black curve) and after (red curve) washing the ultra-thin film in ultra-pure water upon UV cleavage for 1 h.

Before the washing process, the cylindrical structures of the block copolymer are clearly visible both on the topography and on the phase images with a size average of 25 ± 5 nm (calculated from line profiles of the SPM topography image). Regarding the topography image after the XRR measurement, it is conspicuous that the film structure was destroyed. Additionally, the formation of the pores is not given, which can be more clearly seen on the phase image. For the measured SFM images, the bearing analysis was performed to obtain a distribution of the height profiles. This allows for drawing conclusions how far the pores are free. In Figure 4, the two results are shown before and after washing the film. 

We interpret this change as a deposition of the pore material onto the sample surface, as an increase in roughening of only 3 nm is too small to be attributed to the cylinder structure, which has an effect on the height distribution of the surface. This supports the result of the XRR measurement that the two polymer blocks could not be fully cleaved at 1 h of UV cleavage and the previous statement that a part of the pore material diffuses onto the surface and contracts the film by interaction with the substrate.

For the sample, which was cleaved with UV radiation for 4 h, the measurements and analysis were carried out in the same manner. In Figure 1c, the reflectivity curve is shown before the washing process.

**Figure 5:**
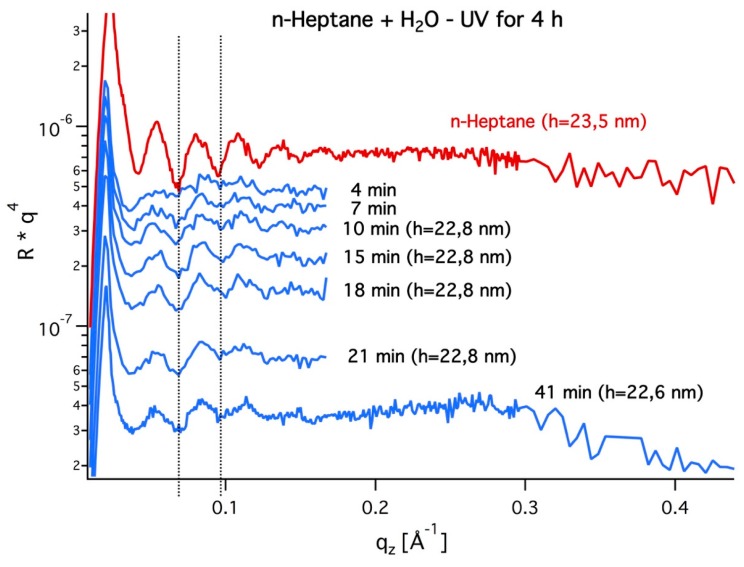
In-situ X-ray reflectivity curves of PS-hν-PEO ultra-thin film UV-cleaved for 4 h during washing out the pore material. The red curve represents the sample in n-heptane for sample alignment. The blue reflectivity curves correspond to the measurements in ultra-pure water with the according washing time and film thickness h. The curves are plotted as $R*q^{4}$ versus $q_{z}$ to eliminate the strong drop of the reflectivity and to enhance the visibility of the Kiessig fringes.

The evaluation of the curve shows a layer thickness of 21.9 nm and roughness of 9 Å for the polymer film. As in the previous sample, an increase in the layer thickness can be also seen after adding *n*-heptane into the liquid cell. The film thickness increased to a value of 23.5 nm (see Figure 5). The reversible process after addition of water is also observed here.

The film thickness in water was 22.8 nm and only decreased minimally during the washing process. Regarding the oscillations, a change in the roughness is observed after 15 min. of washing. This time period is consistent with the first sample, where a time dependent process was observed up to around 12 min. When comparing the measurement in water after 10 min. and 15 min., a weakness of the oscillations at large $q_{z}$ values is also seen in the second sample. This observation persists over the further washing process and is also reflected during the last scan after 41 min., which was measured over a larger angular range. Based on this result, a change in the roughness after washing would be expected, in contrast to the first sample.

Figure 1d shows the proof for this, which shows the reflectivity of the sample after washing and drying for 1.5 h in air. When comparing the obtained parameters, which are listed in Table 2, the roughness after washing is 10 Å and the layer thickness is 21.7 nm. A substantial decrease in the layer thickness could not be established for the sample that was irradiated with UV for 4 h.

**Figure 6:**
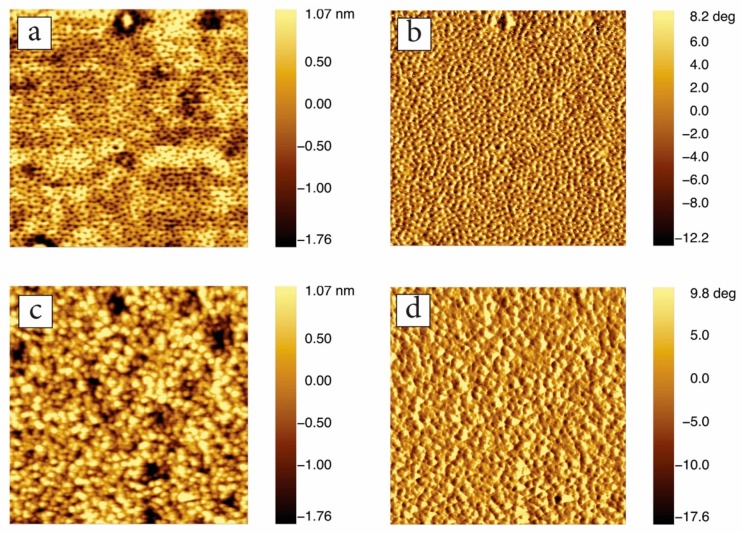
SFM topography (**a**,**c**) and phase (**b**,**d**) images (1μm × 1μm) before (**a**,**b**) and after (**c**,**d**) in-situ measurements with ultra-pure water upon UV cleavage for 4 h.

Therefore, we assume that, with 4 h UV exposure, the cleavage of the two blocks could be achieved and, thus, PEO could be removed from the pores. The SFM recordings were analyzed before and after washing to check this assumption. Figure 6 shows the SFM images of the sample before and after washing. Additionally, in this sample, the loss of the surface structure after washing is clearly visible. However, the formation of the pores can be clearly seen in the phase image (Figure 6d) after the washing process. 

**Figure 7:**
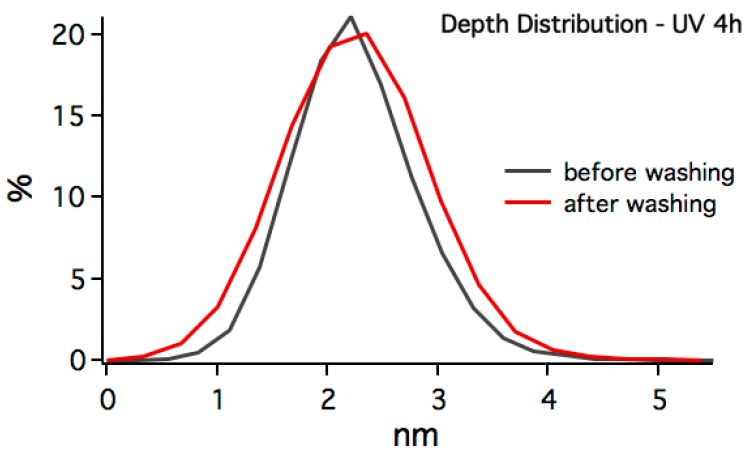
Depth distribution evaluated from the SFM images (Figure 6) before (black curve) and after (red curve) washing the ultra-thin film in ultra-pure water upon UV cleavage for 4 h.

The corresponding bearing analyses were also carried out on these SFM measurements, as shown in Figure 7. When comparing the height profiles, it can be seen that the height of the pores has increased by about 1.5 nm after washing.

**Figure 8:**
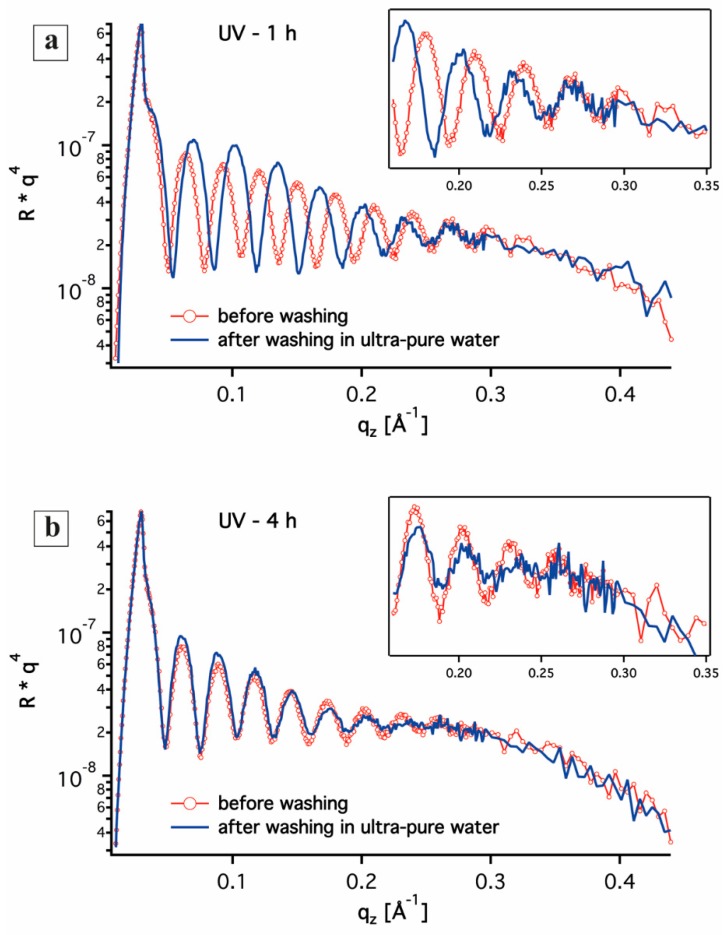
X-ray reflectivity curves of PS-hν-PEO ultra-thin film UV-cleaved for 1 h (**a**) and 4 h (**b**) before (red markers) and after washing in ultra-pure water (blue line). The curves are plotted as $R*q^{4}$ versus $q_{z}$ to eliminate the strong drop of the reflectivity and to enhance the visibility of the Kiessig fringes.

The curves of the ex-situ measurements were also multiplied by $q_{z}^{4}$ for a better visualization of the oscillations and the amplitudes in a similar manner to the reflectivity curves of the in-situ measurements. The curves before and after washing were plotted for each sample in the same plot, as shown in Figure 8. The sample that was irradiated for 1 h with UV shows a shift of the oscillation minima or maxima after the washing when compared to that before washing, which results from a change in the layer thickness (Figure 8a). In contrast, the oscillations of the sample with 4 h UV irradiation show no significant change (Figure 8b). 

Regarding the amplitude of the oscillations, it becomes clear that, for the sample with 1 h of our UV irradiation, the amplitudes are increased after washing. This results from the fact that the electron density in the sample has increased after washing in ultra-pure water. While, the sample with 4 h of UV radiation results in a reduced electron density in the polymer film. 

**Figure 9:**
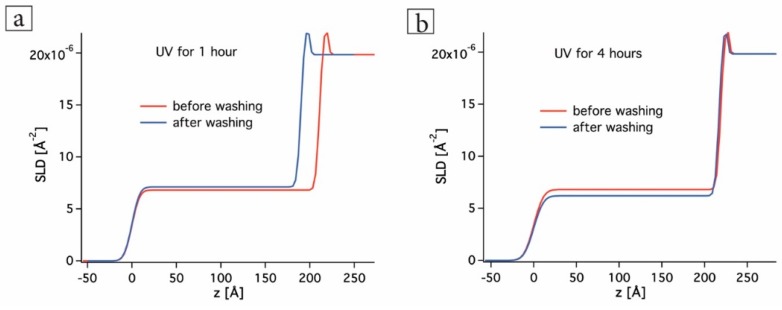
Electron density profiles of PS-*hν*-PEO ultra-thin film UV-cleaved for 1 h (**a**) and 4 h (**b**) before (red lines) and after washing in ultra-pure water (blue lines).

Figure 9 shows the electron density profiles for the respective measurements. The increase in electron density for the sample with 1 h UV irradiation indicates that the pores are not released from PEO. The fact that water is still preserved in the pores leads to an electron density increase. The sample with 4 h UV irradiation gives the opposite result. From this, it can be concluded that the PEO is washed out of the pores and then replaced by water. A quantitative evaluation of the PEO degradation does not allow for reliable statements, since the samples could not be dried under the same conditions after the measurements, thus leading to incomparable water contents after drying. As previously mentioned, the XRR measurements are not sufficient for evaluation of the electron density. Nevertheless, the results that were obtained in this work do not contradict our statements regarding the kinetic behavior of the system.

In summary, one can say that, after 4 h UV irradiation, the two blocks are cleaved, and the pores can be washed with water from PEO. However, the ordered structure of the film is lost. If the time span of 12 or 15 min. determined in the in-situ XRR measurements, it can be defined that the diffusion of the pore material PEO on to the sample surface takes the above-mentioned time. A longer washing process obviously affects the film structure considerably.

### 3.2. Grazing Incidence Small-Angle X-ray Scattering

**Figure 10:**
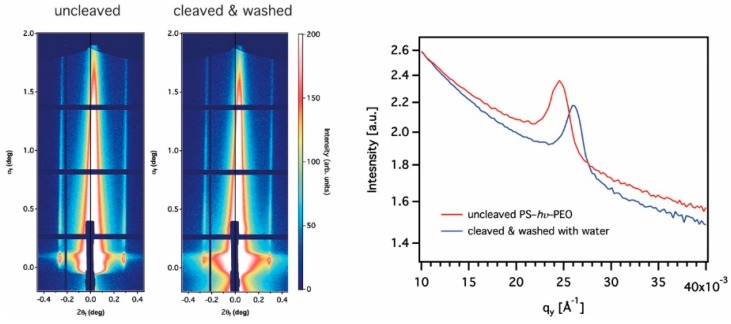
Two-dimensional grazing incidence small-angle X-ray scattering (2D GISAXS) scattering image of PS-*hν*-PEO block copolymer thin film before and after UV cleavage (4 h)/washing with ultra-pure water (50 min.); the high intense symmetric peaks are the first-order diffraction Bragg peaks. Where the plot on the right side is a one-dimensional (1D) out-of-plane profile of the 2D scattering image along the direction of the wavevector $q_{y}$, including both peaks before and after washing with ultra-pure water.

Via GISAXS measurements, the surface morphology of highly ordered block copolymer thin films and the removal of the pore material PEO with ultra-pure water after 4h UV exposure was studied. In Figure 10, the reciprocal space of the 2D ordered PS-*hν*-PEO thin films are shown. The 2D scattering image includes the $q_{z}$ and $q_{y}$ components of the scattering vector perpendicular and parallel to the substrate surface, respectively. The side maxima on the GISAXS 2D image result from the highly lateral ordering and the shape of the scattering peak indicates that a cylindrical formation of the block copolymers exists in the film structure.

The 1D out-of-plane scan cut along the wavevector $q_{y}$ is carried out to extract additional information from the 2D GISAXS data. Figure 10 (right) shows the horizontal intensity cuts of both scattering patterns. On the 1D profile, both scattering peaks can be seen at the $q_{y}$ = 0.02361 Å^−1^ for the uncleaved and $q_{y}$ = 0.02594 Å^−1^ for the cleaved and washed block copolymer thin film. From these parameters, the distance *d* between two domains can be calculated while using the equation:

$$d=\frac{2\;\pi }{q_{y}}$$

For the present measurements, the distance between two domains is 26.6 nm for the uncleaved and 24.2 nm after cleavage and washing. These results show that the PS domains undergo a reorientation after washing with ultra-pure water. As the cylindrical shape of the domains is retained throughout the film after washing, which can be seen in the high intense scattering image after cleavage and washing, the reorientation results from the removal of the PEO from the pores. This is due to the relaxation of the PS domains after the pores are released from the pore material. The GISAXS results are in good agreement with our previous investigation [38].

### 3.3. In-Situ Sacnning Force Microscopy

The in-situ SFM measurements were performed to investigate the influence of the pore material swelling on the surface structure in real time. In addition to in-situ XRR, this measurement provides information regarding the swelling process, which contributes to a better understanding of the kinetics. In Figure 10, the SFM images of the polymer film are shown in the ultra-pure water. The image was started to record from the fourth minute after the polymer film was immersed in water and was then performed in the trace measurement from top to bottom, as well as inversely in the retrace direction.

**Figure 11:**
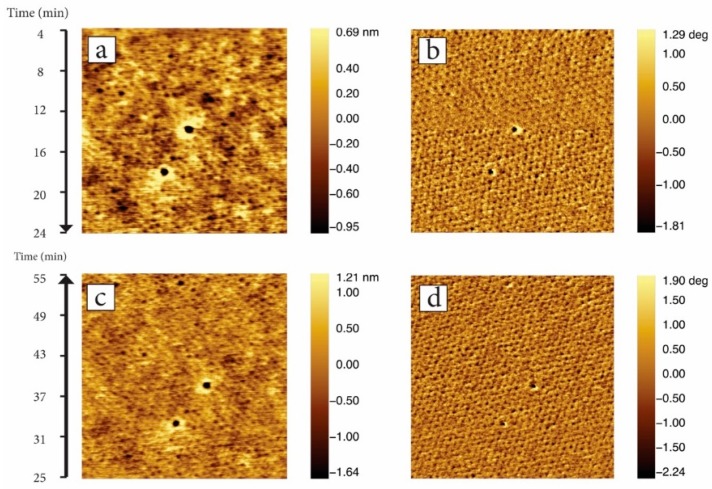
In-situ SFM topography (**a**,**c**) and phase (**b**,**d**) images (1μm × 1μm) during washing with ultra-pure water for 55 min. The recording started 4 min. after immersing the ultra-thin film in ultra-pure water. The trace scan (**a**,**b**) proceeded from top to bottom and the retrace scan (**c**,**d**) in the opposite from bottom to top. UV cleaved the polymer film for 4 h prior to the in-situ recording.

**Figure 12:**
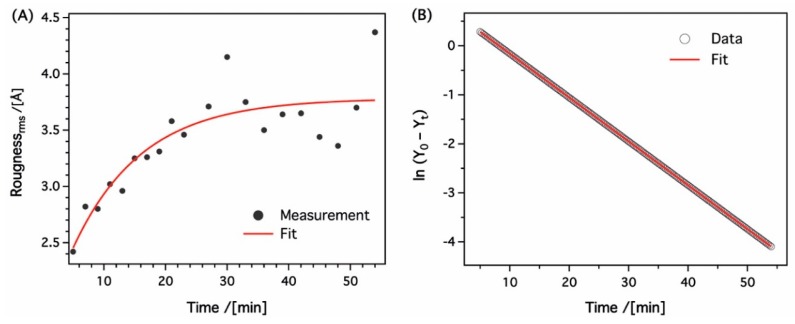
Kinetic evaluation of the washing process based on the surface roughness change of the ultra-thin film during in-situ SFM measurement (Figure 11). The root mean square roughness rms is plotted against washing time (**A**), while (**B**) is the logarithmic plot versus washing time calculated from the exponential function obtained from (**A**) in order to extract the rate constant.

In the initial stage of the measurement, in the range of 0.0–0.2 μm (Figure 11a,b), the highly ordered structure of the film is clearly visible. After a washing time of 10 min., the phase image (Figure 11b) shows how the structure becomes indistinct with increasing time. This indicates that the pore material PEO is rinsed onto the film surface within the period of 15 min. and, thus, the pores are liberated. In addition, it is clear in the image after 15 min., the blurred surface suddenly becomes clearer again, and pores are clearly visible. Looking at the further course of the measurement, a reorientation of the microdomains is observed after approximately 20 min. of washing time. As a result, the hexagonal structure of the film is increasingly destroyed with increasing thee washing time. The changes in the surface structure can be clearly observed in the retrace recording (Figure 11c,d) from the minute 25 and also in the following. This process has an effect on the surface roughness of the film during the measurement, which could be shown in the XRR measurements. The dynamic of the washing process was also quantified via the change in the surface roughness over time, certain sections on the images were selected in the chronology, and the mean square roughness (RMS) parameter was determined. The obtained values were plotted as a function of the washing time, as shown in Figure 12.

The plot in Figure 12A shows that the roughness exponentially increases with washing time. This change in the surface roughness might be related to the formation of the porous structure. No significant increase in the surface roughness beyond 30 min. was observed, indicating that this time is sufficient for the washing process. However, to extract the rate constant of the roughness change and, hence, the washing process, the time dependent of the exponential function obtained by fitting the RMS data in Figure 12A was subjected to linear form of pseudo first order model while using the Lagergren’s pseudo first order kinetic equation:

$$Y_{t}=Y_{0}\left(1-e^{-kt}\right)\;ln\left(Y_{0}-Y_{t}\right)=lnY_{0}-\left(\frac{k}{2.303}\right)t$$

where $Y_{t}$ and $Y_{0}$ are the roughness data at washing time t (min.) and at roughness saturation, respectively. The value of roughness saturation was obtained from the exponential function parameter of nonlinear form of pseudo first order model. While k is the rate constant (min.^–1^). The linearized expression for pseudo first order kinetic model has been shown in the form of a plot of $ln\left(Y_{0}-Y_{t}\right)$ vs t in Figure 12B. The rate constant was determined from the slope of the curve to be 0.21 min.^–1^. It should be noted that a nonlinear relationship between $lnY_{t}$ and t was obtained, indicating that the change in the roughness and, hence, the washing process does not follow the first order kinetic model.

In our previous work, it was shown that both the washing solvent and the washing time significantly affect the structure of the entire film [25]. If the results of the washing protocols are additionally taken into consideration, namely that the speed is considerably faster when ethanol is used as the washing solution, it can be concluded that the kinetics depend on the kind of the solvent in the present system. This means that the affinity of the pore material to the solvent is the determining factor in the washing process.

**Figure 13:**
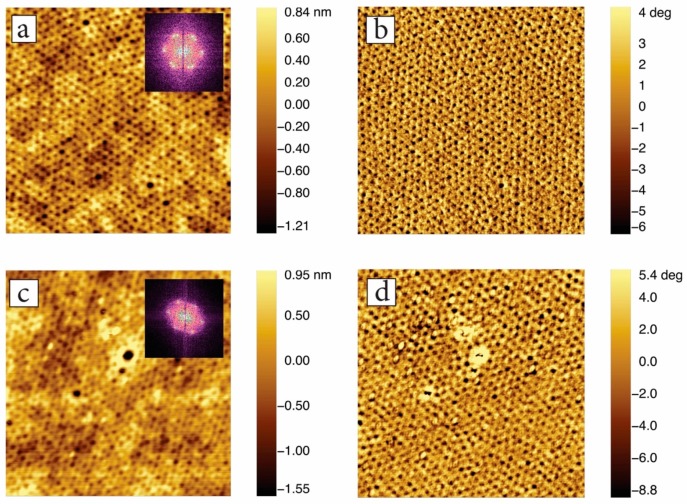
SFM topography (**a**,**c**) and phase (**b**,**d**) images (1μm × 1μm) before (**a**,**b**) and after (**c**,**d**) washing with ultra-pure water for 55 min. from the same sample as used for the in-situ SFM measurements. The insets in (**a**,**c**) show the according FFT analysis.

In addition, SFM was utilized to measure the sample before and after the in-situ investigation in a dry state for a better illustration the changes in the surface structure of the polymer film. Figure 13a,b show the SFM images before washing the film.

The film shows a highly oriented structure in both topography and the phase image, and the cylinders are clearly visible. The FFT analysis, which is shown in the inset of the topography image, gives a hexagonal structure. When comparing both SFM measurements before and after to in-situ investigation, it become apparent that the surface structure is deformed after washing in ultra-pure water. In addition, it can be seen that the FFT analysis for the topography image after washing (Figure 13c) proves an arrested distorted hexagonal arrangement. From this, the conclusion can be drawn that the observed reorientation of the film during the washing process is not reversible and it can also be found in a dry state.

## 4. Summary

The film thickness and roughness of highly ordered photocleavable block copolymer films and the kinetic of PEO block removal at different washing times for ultra-pure water solution was studied via a set of internally consistent XRR measurements and SFM. From the in-situ reflectivity curves, one can conclude that the film thickness as well as the roughness increase after the injection of *n*-heptane into the sample cell, even though *n*-heptane is not a good solvent for both PS and PEO. After *n*-heptane is exchanged with ultra-pure water in the sample cell, we can observe that the swelling is reversible, and the film thickness decrease with increasing washing time. Furthermore, one can see that, after 10 min. washing in water, the film becomes smooth and, after 15 min., the roughness increases again. This can be traced back to the removal of the pore material PEO, which goes into solution after 15 min. washing with ultra-pure water. After drying and scanning the sample in air, the distinct change in the roughness can be shown in the reflectivity analysis. The obtained results were confirmed by the in-situ SFM measurements and the evaluation of the SFM images with regard to the surface roughness of the polymer film. From the surface roughness change during the washing process, a pseudo first order model was found to represent the kinetic and the rate constant was also determined. Furthermore, the GISAXS data clearly showed the formation of the porous structure of the polymer thin film after 4 h UV cleavage and 50 min. washing with water.

## Figures and Tables

**Table 1:** X-ray reflectivity (XRR) data obtained from the measured curve for PS-*hν*-PEO thin film in Figure 1a,b. The polymer film was cleaved for 1 h with UV and immersed for 41 min. in ultra-pure water.

Sample	Roughness [Å]	Film Thickness [nm]	Electron Density [Å^−2^]
Uncleaved	6.7	21.9	6.802 × 10^−6^
PS-*hν*-PEO			
Cleaved PS-*hν*-PEO	6.8	19.0	7.102 × 10^−6^
41 min. H_2_O washing			

**Table 2:** XRR data for PS-*hν*-PEO thin film obtained from the measured curves in Figure 1c,d. The polymer film was UV cleaved for 4 h and immersed for 41 min. in ultra-pure water.

Sample	Roughness [Å]	Film Thickness [nm]	Electron Density [Å^−2^]
Uncleaved	9	21.9	6.802 × 10^−6^
PS-*hν*-PEO			
Cleaved PS-*hν*-PEO	10	21.7	6.202 × 10^−6^
41 min. H_2_O washing			

## References

[1] Gin D.L., Gu W. (2001). Nanoporous Catalytic Materials with Organic Frameworks. Adv. Mater..

[2] Davis M.E. (2002). Ordered porous materials for emerging applications. Nature.

[3] Lei C., Shin Y., Liu J., Ackerman E.J. (2002). Entrapping Enzyme in a Functionalized Nanoporous Support. J. Am. Chem. Soc..

[4] Costeux S. (2014). CO_2_-Blown Nanocellular Foams. J. Appl. Polym. Sci..

[5] Guo H., Nicolae A., Kumar V. (2015). Solid-State Microcellular and Nanocellular Polysulfone Foams. J. Poly. Sci. Part B.

[6] Krause B., Koops G.-H., van der Vegt N., Wessling M., Wübbenhorst M., van Turnhout J. (2002). Ultralow-k Dielectrics Made by Supercritical Foaming of Thin Polymer Films. Adv. Mater..

[7] Jang J., Bae J. (2005). Fabrication of mesoporous polymer using soft template method. Chem. Commun..

[8] Segalman R.A. (2005). Patterning with block copolymer thin films. Mater. Sci. Eng..

[9] Bosworth J.K., Ober C.K. (2012). Top-Down versus Bottom-Up Patterning of Polymers. Polym. Sci. A Compr. Ref..

[10] Bang J., Jeong U., Ryu D.Y., Russell T.P., Hawker C.J. (2009). Block Copolymer Nanolithography: Translation of Molecular Level Control to Nanoscale Patterns. Adv. Mater..

[11] Wu D., Xu F., Sun B., Fu R., He H., Matyjaszewski K. (2012). Design and Preparation of Porous Polymers. Chem. Rev..

[12] Ochsmann J.W., Lenz S., Emmerling S.G.J., Kappes R.S., Nett S.K., Lechmann M.C., Roth S.V., Gutmann J.S. (2010). PS-b-PEO block copolymer thin films as structured reservoirs for nanoscale precipitation reactions. J. Polym. Sci. Part B Polym. Phys..

[13] Hong S.W., Russell T.P. (2012). Block Copolymer Thin Films. Polym. Sci. Compr. Ref..

[14] Hamley I.W. (2009). Ordering in thin films of block copolymers: Fundamentals to potential applications. Prog. Polym. Sci..

[15] Yang S.Y., Ryu I., Kim H.Y., Kim J.K., Jang S.K., Russell T.P. (2006). Nanoporous Membranes with Ultrahigh Selectivity and Flux for the Filtration of Viruses. Adv. Mater..

[16] She M.-S., Lo T.-Y., Hsueh H.-Y., Ho R.-M. (2013). Nanostructured thin films of degradable block copolymers and their applications. NPG Asia Mater..

[17] Hacking S.A., Du Y. (2012). Patterning of Polymeric Materials for Biological Applications. Polym. Sci. Compr. Ref..

[18] Li M., Douki K., Goto K., Li X., Coenjarts C., Smilgies D.-M., Ober C.K. (2004). Spatially Controlled Fabrication of Nanoporous Block Copolymers. Chem. Matter..

[19] Du P., Li M., Douki K., Li X., Garcia C.B.W., Jain A., Smilgies D.-M., Fitters L.J., Gruner S.M., Wiesner U. (2004). Additive-Driven Phase-Selective Chemistry in Block Copolymer Thin Films: The Convergence of Top-Down and Bottom-Up Approaches. Adv. Matter.

[20] Xu T., Goldbach J.T., Misner M.J., Kim S., Gibaud A., Gang O., Ocko B., Guarini K.W., Black C.T., Hawker C.J. (2004). Scattering Study on the Selective Solvent Swelling Induced Surfcae Reconstruction. Macromolecules.

[21] Leiston-Belanger J.M., Russell T.P., Drockenmuller E., Hawker C.J. (2005). A Thermal and Manufacturable Approach to Stabilized Diblock Copolymer Templates. Macromolecules.

[22] Lee J.S., Hirao A., Nakahama S. (1988). Polymerization of monomers containing functional silyl groups. 5. Synthesis of new porous membranes with functional groups. Macromolecules.

[23] Bang J., Kim S.H., Drockenmuller E., Misner M.J., Russell T.P., Hawker C.J. (2006). Defect-Free Nanoporous Thin Films from ABC Triblock Copolymers. J. Am. Chem. Soc..

[24] Kang M., Moon B. (2009). Synthesis of Photocleavable Poly(styrene-block-ethylene oxide) and Its Self-Assembly into Nanoporous Thin Films. Macromolecules.

[25] Schumers J.M., Vlad A., Huynen I., Gohy J.F., Fustin C.A. (2012). Functionalized nanoporous thin films from photocleavable block copolymers. Macromol. Rapid Commun..

[26] Zhao H., Gu W., Sterner E., Russell T.P., Coughlin E.B., Theato P. (2011). Highly Ordered Nanoporous Thin Films from Photocleavable Block Copolymers. Macromolecules.

[27] Müller-Buschbaum P. (2008). Chapter 2: Structure Determination in thin Film Geometry Using Grazing Incidence Small-Angle Scattering. Polymer Surfaces and Interfaces.

[28] Müller-Buschbaum P., Hermsdorf N., Roth S.V., Wiedersich J., Cunise S., Gehrke R. (2004). Comparative analysis of nanostructured diblock copolymer films. Spectrochim. Acta Part B.

[29] Tolan M. (1999). X-Ray Scattering from Soft-Matter Thin Films: Materials Science and Basic Research.

[30] Pietsch U., Holy V., Baumbach T. (2004). High-Resolution X-Ray Scattering: From Thin Films to Lateral Nanostructures.

[31] Wirkert F.J., Paulus M., Nase J., Möller J., Kujawski S., Sternemann C., Tolan M. (2014). X-ray reflectivity measurements of luquid/solid interfaces under high hydrostatic pressure conditions. J. Synchrotron Radiat..

[32] Kiesel I., Paulus M., Nase J., Tiemeyer S., Sternemann C., Rüster K., Wirkert F.J., Mende K., Büning T., Tolan M. (2014). Temperature-driven Adsorption and Desorption of Proteins at Solid-Liquid Interfaces. Langmuir.

[33] Wirkert F.J., Paulus M., Sternemann C., Nase J., Schroer M.A., Wieland D.C.F., Bieder S., Degen P., Rehage H., Tolan M. (2013). Study of time and pressure dependent phenomena at the hard x-ray beamline BL9 of DELTA. J. Phys. Conf. Ser..

[34] Paulus M., Lietz D., Sternemann C., Shokuie K., Evers F., Tolan M., Czeslik C., Winter R. (2008). An access to buried interfaces: The X-ray reflectivity set-up of BL9 at DELTA. J. Synchrotron Radiat..

[35] Hexemer A., Bras W., Glossinger J., Schaible E., Gann E., Kirian R., MacDowell A., Church M., Rude B., Padmore H. (2010). A SAXS/WAXS/GISAXS Beamline with Multilayer Monochromator. J. Phys. Conf. Ser..

[36] Babonneau D. (2010). Software package for modelling and analysis of GISAXS data using IGOR Pro. J. Appl. Crystallogr..

[37] Hansen C.M. (2007). Hansen Solubility Parameters: A User’s Handbook.

[38] Altinpinar S., Zhao H., Ali W., Kappes R., Schuchardt P., Salehi S., Santoro G., Theato P., Roth S., Gutmann J.S. (2015). Distortion of Ultrathin Photocleavable Block Copolymer Films during Photocleavage and Nanopore Formation. Langmuir.

